# The Impact of Power on Humanity: Self-Dehumanization in Powerlessness

**DOI:** 10.1371/journal.pone.0125721

**Published:** 2015-05-28

**Authors:** Wenqi Yang, Shenghua Jin, Surina He, Qian Fan, Yijie Zhu

**Affiliations:** School of Psychology, Beijing Normal University, Beijing, P. R. China; Cardiff University, UNITED KINGDOM

## Abstract

Power gives people the ability to control themselves and their environment, and this control is considered a fundamental human need. We investigated whether experiencing powerlessness induces the experience of self-dehumanization using three methods: priming, role-playing, and cueing. People in a position of low power viewed themselves (Experiments 1–3) as less human relative to people in a position of high power; furthermore, people with low power believed that they were viewed as less human by others as well (Experiments 2–3). In all of the experiments, human nature traits were most negatively affected by powerlessness in self-perception judgments, and uniquely human traits were most negatively affected by powerlessness in meta-perception judgments. Furthermore, the powerless believed they were viewed as less human not only by the powerful people but also the outside observers of the power dynamic. Self-dehumanization also appears to be a consequence of powerlessness rather than an incidental result of a change in mood or a negative self-view. Our findings are an important extension of previous work on the adverse effects of powerlessness and dehumanization.

## Introduction

Power allows people to control outcomes with respect to both the environment and the self [1–5]. This control is considered to be a fundamental human need [6, 7]; therefore, it follows that powerlessness will disrupt an individual’s sense of humanity, which coincides with the fact that many civilizations have equated power deprivation with humbleness, lowliness, and fewer expressed human traits [8]. We based the current study on this notion in order to demonstrate that perceived powerlessness induces self-dehumanization.

### Self-dehumanization

The central feature of dehumanization is a failure to attribute feelings or qualities of mind to humans [9–13]. Human nature (HN) traits are attributes that are biologically based, innate, shared, fundamental, or essential features of humanity. They are associated with cognitive flexibility, emotional reactivity, agency, openness, and individuality [10]. Attributing a lack of HN traits to people is akin to explicitly or implicitly perceiving or acting toward those people as though they lack the capacity to feel (i.e., as if they were automatons). In contrast, uniquely human (UH) traits are acquired through education and interaction with one’s culture, are distinctive to the human species, and are associated with refinement, responsibility, maturity, enlightened morality, civility [10], and higher cognition [13]. Attributing a lack of UH traits to people is akin to explicitly or implicitly perceiving or acting toward those people as though they lack the capacity to think (i.e., as if they were non-human animals). In other words, this framework implies that people can be dehumanized in two ways: mechanistically (via a lack of HN traits) or animalistically (via a lack of UH traits). To date, self-dehumanization, in addition to being rarely examined, has been studied only in terms of behaviors related to the immoral treatment of the self or others [14–16]. The present study aimed to extend previous findings by assessing ordinary interpersonal situations in which power and self-perceived humanity are involved.

People can see themselves in two ways: they can adopt a first-person perspective, wherein they see themselves through their own eyes (self-perception), or they can adopt a third-person perspective, wherein they see themselves through others’ eyes (meta-perception) [17]. Theoretically, people experience self-dehumanization not only when they perceive themselves as less human but also when they believe that others attribute less human traits to them. Surprisingly, researchers have studied self-dehumanization only from a self-perception perspective [15–16]; there is little direct evidence to support a meta-perception perspective, except for one study that investigated meta-perception in social ostracism [14]. As such, we investigated self-dehumanization of powerless individuals from both perspectives.

### Powerlessness and self-dehumanization

Recent work has begun to establish more links between powerlessness and dehumanization—specifically, the ways in which powerlessness catalyzes the process of dehumanization among powerless individuals [18–20]. However, it is unclear whether perceived powerlessness has consequences for how we see ourselves, particularly with respect to self-dehumanization. Thus, the current study examined the relationship between feelings of powerlessness and self-dehumanization. Previous research suggests that the two types of human traits (HN and UH) contribute to self-perception and meta-perception within the context of interpersonal maltreatment [14, 21]. Accordingly, we argue that both mechanistic and animalistic dehumanization are related to powerlessness.

Self-perception directs a person’s attention inward, focusing on the self and comparing it with the most salient self-relevant standard or goal [22]. HN traits comprise characteristics that are typically or essentially human—that is, traits that represent the “core” of a human [10] and occur in the context of interactions in which people “disregard the existence of other people as social partners” [23]. If mechanistic dehumanization represents such a lack of relatedness perception, it would understandably be apparent in self-perception. This is also consistent with the notion that when people feel they are treated like objects (a denial of HN) they report a range of states of cognitive deconstruction (e.g., emotional numbing) [14]. Therefore, we expect that when people are in powerless positions, they attribute fewer HN traits to themselves during self-perception. That is, such individuals perceive themselves as mechanical, rather than as complete human beings. This notion has been implied in research examining the experiential impact of powerlessness on self-perception, particularly to the notion of the “constrain state,” which is characterized by a lack of freedom and one’s behavior being dictated by others or the environment as opposed to one’s own will [24, 25], as well as impaired cognitive flexibility and selectivity [26, 27]. As agency, freedom, and cognitive flexibility are all qualities commonly thought to be central to human nature [10, 13], powerlessness may cause people to feel as if they possess fewer HN traits.

In contrast, meta-perception focuses a person’s attention on the external environment (e.g., tasks, other people, scenery), wherein that person compares the self with externalized standards [22]. UH traits might be perceived as nonessential, in that they reflect socialization and culture and are restricted by the context [10]. If animalistic dehumanization represents perceiving a lack of such traits in interpersonal contexts, it would understandably be apparent in meta-perception. This is consistent with the notion that the experience of being treated like an animal (lacking UH traits) is strongly related to self-consciousness and emotions such as shame [20], which are thought to signify a loss of social status or social value and feelings of inferiority [28, 29]. Therefore, we expect that when people are powerless, they attribute fewer UH traits to themselves during meta-perception. That is, such individuals consider themselves animalistic entities, rather than complete human beings, in others’ eyes. This is has been implied by research suggesting that powerless individuals have few or no opportunities to express their abilities and intelligence [30, 31] and have less of an ability to focus on primary goals [32]; as such, they tend to be considered less kind and less moral by others [33]. These effects suggest that powerless individuals are considered to lack maturity, rationality, responsibility, self-control, and trustworthiness, which are generally considered predominant UH traits [10, 13]; thus, powerless individuals tend to be perceived as having fewer UH traits, rendering them more animalistic.

The purpose of the present study was to investigate the relationships of experiencing powerlessness with perceiving oneself as less human and being viewed as such by others (i.e., the powerful and observers outside the power dynamic), while controlling for the mood and negative self-perceptions that often accompany powerlessness. We also predicted that the two types of dehumanization might have different effects on self-perception and meta-perception, according to the definitions of the two types of dehumanization and their relationships with power. Specifically, we predicted that powerlessness leads people to attribute fewer HN traits in self-perception and fewer UH traits in meta-perception.

## Overview

We conducted three experiments to test the following hypotheses: (1) Powerless individuals will tend to show a reduction in self-perceived (self-perception and meta-perception) humanity; (2) experiencing low power will decrease HN traits in self-perception and UH traits in meta-perception; and (3) the effects of self-dehumanization due to powerlessness would be independent of a negative self-view and mood changes.

In Experiment 1, we used a priming manipulation directly related to power and assessed whether it would induce self-dehumanization in powerless individuals. In Experiment 2, we used role-playing manipulations to extend our findings and assess whether individuals in positions of low power are more likely to engage in self-dehumanization. In Experiment 3, we deployed a subtler method to conceptually replicate and extend the findings of Experiment 2, testing whether environmental cues can activate the concept of power and further investigating the self-dehumanizing effect of low power. Our prediction for all experiments was that individuals with low power would consider themselves less human and would believe themselves to be viewed as such by others, independent of any accompanying negative self-views and mood changes. Specially, we also predicted that low power would affect HN traits in self-perception and UH traits in meta-perception, in accordance with the rationale provided in the preceding section.

## Experiment 1

Experiment 1 examined the impact of power on self-perception of humanization. We made predictions regarding HN traits.

### Method

#### Ethics Statement

The experiment was conducted after obtaining Institutional Review Board approval from the School of Psychology at Beijing Normal University. All participants gave informed written consent before testing began.

#### Participants

One hundred thirteen adult students (68 female) from Beijing Normal University received course credits in return for their participation in this experiment. Their ages ranged from 21 to 37 years (*M =* 25.89, *SD =* 1.89).

#### Procedure

*Power manipulation*. Participants were randomly assigned to either the high- or the low-power group and instructed to provide a vivid written report of a past event in which they had exercised power over another individual or someone else had exercised power over them, in accordance with the procedure used by Galinsky and colleagues [4]. They were instructed to write for 15–20 minutes, vividly imagine the scenes, and include as many details as possible. All participants used a 20-line, A4-size sheet of paper to write about their experiences. They were asked to fill at least three-quarters of the page. There were no differences between the conditions with respect to the amount of information recalled (lines completed: *M =* 17.3), *t*(102) < 0.5, *p >* .05.

*Dehumanization traits*. Following power priming, participants rated themselves with regard to 12 items, adapted from a study conducted by Haslam [10], to measure HN and UH traits as follows: for HN traits, “I felt like I had interpersonal warmth”; “I felt that I was emotional, like I was responsive and warm”; “I felt like I was open minded, like I could think clearly about things”; “I felt like I was an object, not a human” [reversed]; “I felt like I was mechanical and cold, like a robot” [reversed]; “I felt superficial, like I had no depth” [reversed]; and for UH traits, “I felt like I was refined and cultured”; “I felt like I was an adult, not a child”; “I felt like I was rational and logical, like I was intelligent”; “I felt like I lacked self-restraint, like an animal” [reversed]; “I felt like I was unsophisticated” [reversed]. Participants responded using a 7-point scale, ranging from 1 (not at all) to 7 (very much so). HN and UH indices were constructed by reverse scoring the reversed items, and then adding these scores to the scores of the other items. Lower scores indicated greater feelings of dehumanization—as such, we expected participants in the low-power condition to have lower scores than those in the high-power group.

*Affect*. Next, participants used a 7-point scale (1 = very negative to 7 = very positive) to rate how they felt at that particular moment. Previous research has shown that a lack of power may induce negative affect [4], which may accompany the process of dehumanization.

Participants completed the task in a classroom setting; the process lasted approximately 30 minutes. Participants were then probed as to their understanding of the purpose of the study, debriefed, and thanked individually.

### Results

Nine participants were excluded. Two were unwilling to recall relevant experiences, two did not complete the writing task adequately, two had just participated in a similar priming procedure, and three expressed suspicion regarding the purpose of the experiment. Our analysis was conducted using the data from the remaining 104 participants.

#### Manipulation check

Consistent with previous research [34], two independent judges (blind to the experimental conditions) rated the essays using a 7-point scale (1 = no power at all, 7 = rich in power) to determine how much power participants appeared to have exercised in their descriptions in the power-priming essays (r = .88). We used the mean score for coders’ ratings. As expected, the judges rated participants in the high-power condition as significantly more powerful (*M* = 5.94, *SD* = 0.57) than did those in the low-power condition (*M* = 2.01, *SD* = 0.74), *t*(102) = 30.47, *p* < .001.

#### Affect

High-power participants (*M =* 5.10, *SD =* 1.05) rated affect more positively than did those who were in the low-power condition (*M =* 3.69, *SD =* 1.15), *t*(102) = 6.50, *p <* .001.

#### Dehumanization traits

The measures of sense of humanity were reliable (HN: Cronbach’s *α* = .72; UH: Cronbach’s *α* = .68). The self-perception scores for the two types of human trait were averaged separately across participants and then subjected to a repeated-measures ANCOVA. Power (high vs. low) was the between-subjects factor and was considered a test of the study’s main hypothesis (i.e., lower attribution of human traits in the low-power condition), while trait (HN vs. UH) was the within-subjects factor. Affect ratings and gender were assigned as covariates in the repeated-measures ANCOVA, as previous research has shown that gender may affect sense of power [35].

**Fig 1:**
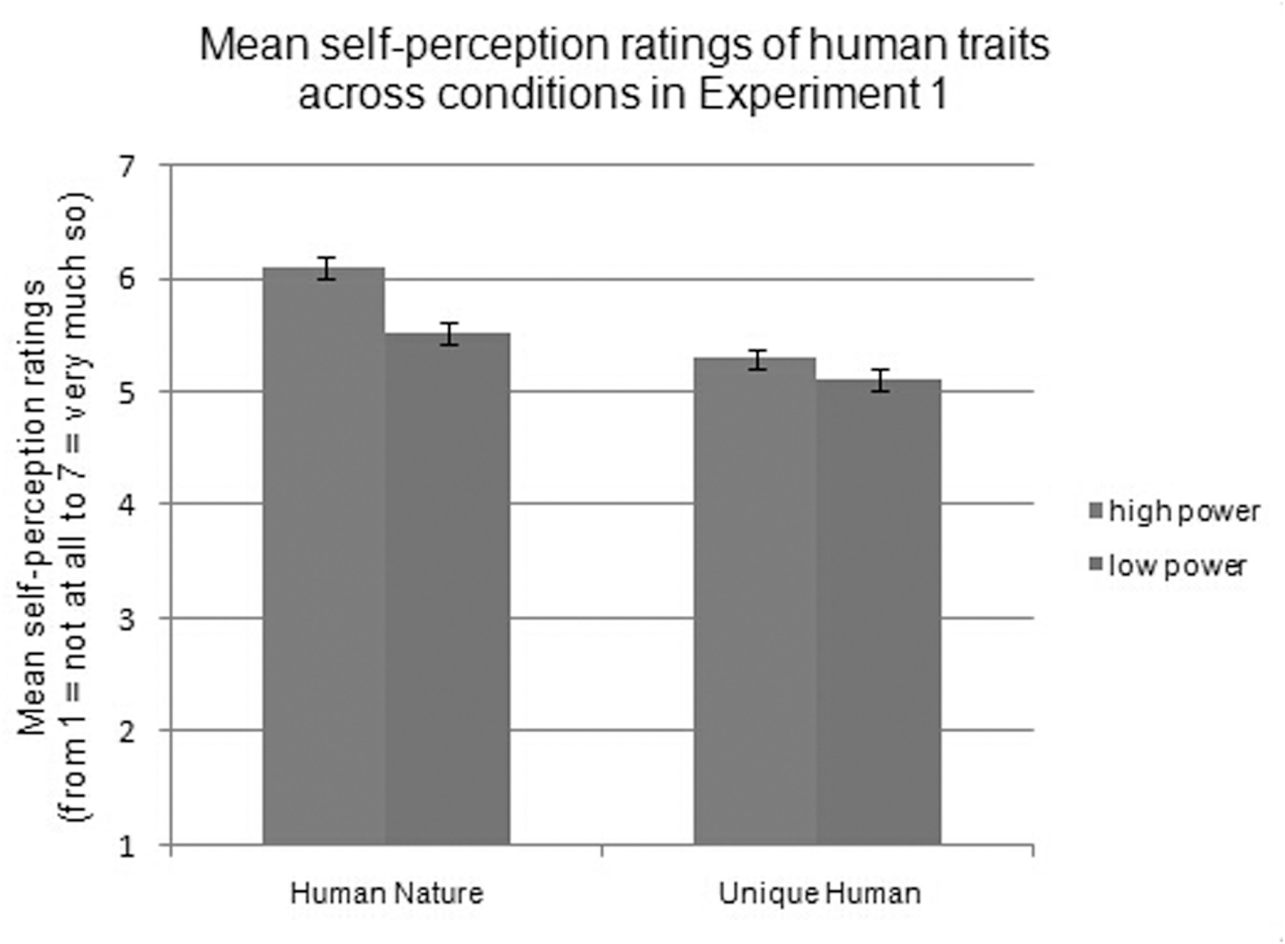
Mean self-perception ratings of human traits across conditions in Experiment 1.

In line with our predictions, the experimental manipulation of power significantly affected participants’ self-dehumanization, *F*(1, 100) = 12.19, *p* = .001, ƞ^2^ = .11. Specifically, without considering the specific types of human traits, participants in the low-power condition rated themselves as having significantly fewer human traits (*M* = 5.29, *SD =* 0.60) than did those in the high-power condition (*M =* 5.74, *SD =* 0.48). Trait interacted with power, *F*(1, 100) = 8.61, *p* = .004, ƞ^2^ = .08. Simple effects analysis showed that participants in the low-power condition rated themselves as having fewer HN traits than did those in the high-power condition (*M*_high-power_ = 6.11; *M*_low-power_ = 5.52; *t*(100) = 18.11, *p <* .001, ƞ^2^ = .15); however, UH traits were similar across power conditions (*M*_high-power_ = 5.31; *M*_low-power_ = 5.11; *t*(100) = 2.64, *p* = .107), as shown in Fig 1.

### Discussion

The first experiment provided initial evidence for a link between powerlessness and reduced self-perceived humanity. Support was also found for the importance of HN traits, with lower power negatively affecting the self-perceived sense of shared humanity (HN traits). Notably, we did not find any effect of power on self-perceived UH traits. Moreover, the first experiment showed that the self-dehumanizing effect of powerlessness was independent of the negative affect that may accompany dehumanization. Experiment 1 did, however, leave several questions unanswered.

First, by relying solely on recall manipulation, it was impossible to control for other factors that were irrelevant to the study but that may have led to the participants’ reduced self-perceived humanity. Second, all of the HN trait items were positive. As such, rather than specifically reflecting a loss of a sense of humanity, our findings may be explained by unrelated negative self-perceptions. A further question that arose was about how participants believed they were viewed by powerful individuals. The literature on meta-perception suggests that people have accurate knowledge of how others perceive them [17]. That is, although people are not particularly adept at knowing what particular individuals think of them, they do have an accurate sense of how most individuals perceive them [36].

## Experiment 2

In the second experiment, we sought to replicate and solidify the results found in Experiment 1 by using role-play to prime power along with different measures of human traits to eliminate the influences of negative self-view as well as mood. We predicted that powerless would lead participants to not only feel less human, but also believe that powerful individuals would view them as such. Specifically, we predicted that powerlessness leads people to attribute fewer HN traits in self-perception and fewer UH traits in meta-perception.

### Method

#### Ethics Statement

The experiment was conducted after obtaining Institutional Review Board approval from the School of Psychology at Beijing Normal University. All participants gave informed written consent before testing began.

#### Participants

Eighty-eight adults (48 female) received 15 RMB in return for their participation in this experiment. Their ages ranged from 22 to 35 years (*M =* 28.57, *SD =* 1.83).

#### Procedure

*Power manipulation*. The role-based procedure for manipulating power was adapted from Galinsky and colleagues [4] and Anderson and Berdahl [37]. Participants arrived at the room in pairs and were randomly assigned to either the high-power condition or the low-power condition in same-sex dyads. They were initially required to complete 20 items from the Big Five Inventory [38]. Thereafter, the experimenter informed the participants that they would be required to perform a coordination task wherein they would build a Tanagram from a set of Legos; in this coordination task, one person would take the role of the leader and the other the role of the subordinate. Participants were informed that the roles would be assigned based on their responses to the personality questionnaire, and the experimenter subsequently left the room to score the questionnaires. In fact, the roles of leader and subordinate were randomly assigned. Approximately 5 minutes later, the experimenter returned, announced the results, and described the roles.

Leaders were informed that they could exert complete control over the work, structure and direct the whole process, do whatever they wished, and instruct their subordinates to do what the leaders desired. In addition, leaders were required to evaluate the subordinates at the end of the session via a questionnaire that the subordinates were forbidden from looking at. Leaders determined the evaluation standards and their ultimate evaluation determined how the participants’ fees (RMB 30) were divided between the leader and subordinate in each dyad. Leaders were not evaluated by anyone. In contrast, subordinates were informed that their leaders would decide how to structure and direct the whole process and that they should carry out the task as instructed by the leaders, regardless of their own will. In addition, the subordinates understood that their leaders determined the standards for evaluating the subordinates’ performance at the end of the session. The subordinates could not see the leaders’ evaluations, which would determine how the participants’ fees (RMB 30) would be divided between the leader and subordinate in each dyad. Subordinates would not have an opportunity to evaluate their leaders.

The experimenter then told participants that they needed to leave the room to prepare for building the Tanagram. Then the participants were asked to complete a questionnaire that was described as a pretest for the study. In fact, the questionnaire was the measurement of humanity.

*Dehumanization traits*. Participants rated themselves using 20 items [14] rated on a 7-point scale (1 = not at all to 7 = to a great extent), which included five positive HN traits (active, curious, friendly, helpful, and fun-loving), five negative HN traits (impatient, impulsive, jealous, nervous, and shy), five positive UH traits (broadminded, conscientious, humble, polite, and thorough), and five negative UH traits (disorganized, hard-hearted, ignorant, rude, and stingy). The HN and UH traits have each been shown to epitomize one of the two senses of humanity [11] and both have two positive and two negative valence traits.

Participants also rated meta-perception for the same items, after the item stems were changed to “I felt like the leader participant saw me…(for the powerless participants)” or “I felt like the subordinate participant saw me…(for the powerful participants)”. In particular, they were asked to think about their “experience of power” and to answer each question accordingly.

*Affect*. Next, participants used a 7-point scale (1 = very negative to 7 = very positive) to rate how they felt at that particular moment.

*Manipulation check*. At the end of the procedure, all participants completed two manipulation checks according to the procedure used by Anderson and Berdahl [37]: “Who had more control over the way in which you completed the task?” and “Who was more dominant during your interaction?”. They rated each item from 1 (the opponent) to 7 (me).

Participants completed the task, which took approximately 30 minutes in a separate room. They were then probed to ascertain whether they had any suspicion of the hypothesis and were debriefed individually.

### Results

Eight participants were excluded. Two (male) pairs knew each other before the experiment, two (male) participants expressed some suspicion regarding the purpose of the experiment, and two (male) participants did not complete the self-dehumanization measures properly. The analyses were conducted using the data from the remaining 80 participants.

#### Manipulation check

The two items for the manipulations check were found to be reliable (Cronbach’s α = .83). We used the average score of these items to form an overall sense of power score. As expected, participants in the high-power role described themselves as more powerful (*M* = 5.13, *SD* = 0.99) than did those in the low-power role (*M* = 3.07, *SD* = 0.76), *t*(78) = 10.35, *p* < .001.

#### Affect

No effects of the high-power or low-power condition on overall affect scores were revealed, *ps* > .05, so these data will not be included in the following analysis.

#### Dehumanization traits

The measures of sense of humanity had acceptable reliability (self-perception, HN: Cronbach’s *α* = .71, HU: Cronbach’s *α* = .60; meta-perception, HN: Cronbach’s *α* = .73, HU: Cronbach’s *α* = .67). The self-perception and meta-perception scores for the two types of human traits were averaged separately across participants and subjected to repeated-measures ANCOVAs. As mentioned above, power (high vs. low) was the between-subjects factor and was considered to represent a test of the study’s main hypothesis, while valence (positive vs. negative) and trait (HN vs. UH) were the within-subjects factors. We also entered participant gender as a covariate in the repeated-measures ANCOVA, as in Experiment 1.

**Fig 2:**
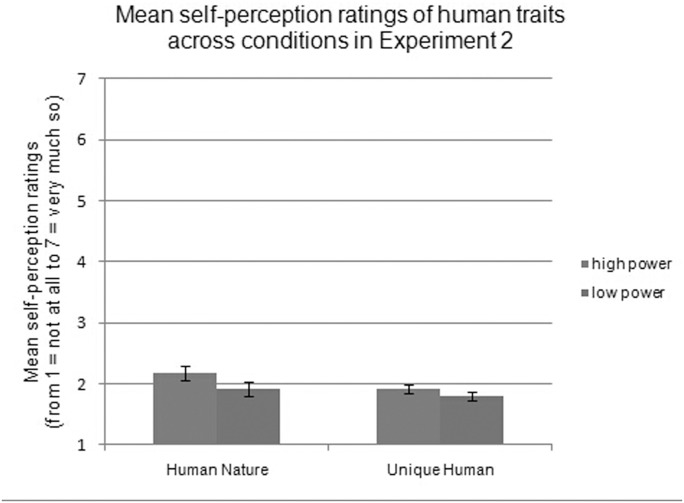
Mean self-perception ratings of human traits across conditions in Experiment 2.

For the ratings of self-perceptions, a significant 2 (power: high vs. low) × 2 (valence: positive vs. negative) × 2 (trait: HN vs. UH) interaction effect was not found, *F*(1, 77) = 2.98, *p* = .088. Then, we conducted separate 2 (power: high vs. low) × 2 (valence: positive vs. negative) repeated-measures ANCOVAs for each type of human trait. For HN traits, a main effect of valence was found, *F*(1, 77) = 24.07, *p <* .001, ƞ^2^ = .24, revealing that participants rated themselves as having more positive (*M =* 2.47, *SD* = 0.76) than negative (*M =* 1.61, *SD* = 0.63) HN traits. HN traits also showed a main effect of power, *F*(1, 77) = 6.03, *p* = .016, ƞ^2^ = .07, demonstrating that participants who lacked power viewed themselves as having fewer HN traits (*M* = 1.91, *SD* = 0.74) than did those who had power (*M* = 2.17, *SD* = 0.74), as shown in Fig 2. This supports our hypothesis concerning the importance of perceived power in the expression of HN traits. Valence interacted with power, *F*(1, 77) = 9.38, *p* = .003, ƞ^2^ = .11, and the simple effects analysis showed that people who lacked power rated themselves as having fewer positive HN traits while simultaneously ascribing equal numbers of negative HN traits to themselves compared to those who had power (positive: *t*(77) = 12.59, *p* = .001, ƞ^2^ = .14; negative: *t*(77) = .07, *p* = .795).

For the UH dimension, there was a robust main effect of valence, *F*(1, 77) = 56.38, *p <* .001, ƞ^2^ = .42, with participants rating themselves as having more positive (*M =* 2.55, *SD =* .55) than negative (*M =* 1.19, *SD =* .50) UH traits. However, mean UH ratings did not differ across power conditions, *F*(1, 77) = 2.92, *p* = .092, demonstrating that participants who lacked power had equal numbers of UH traits (*M =* 1.81, *SD =* .45) as those who had power (*M =* 1.92, *SD =* .45), as shown in Fig 2. Valence did not interact with power, *F*(1, 77) = 2.97, *p* = .089.

**Fig 3:**
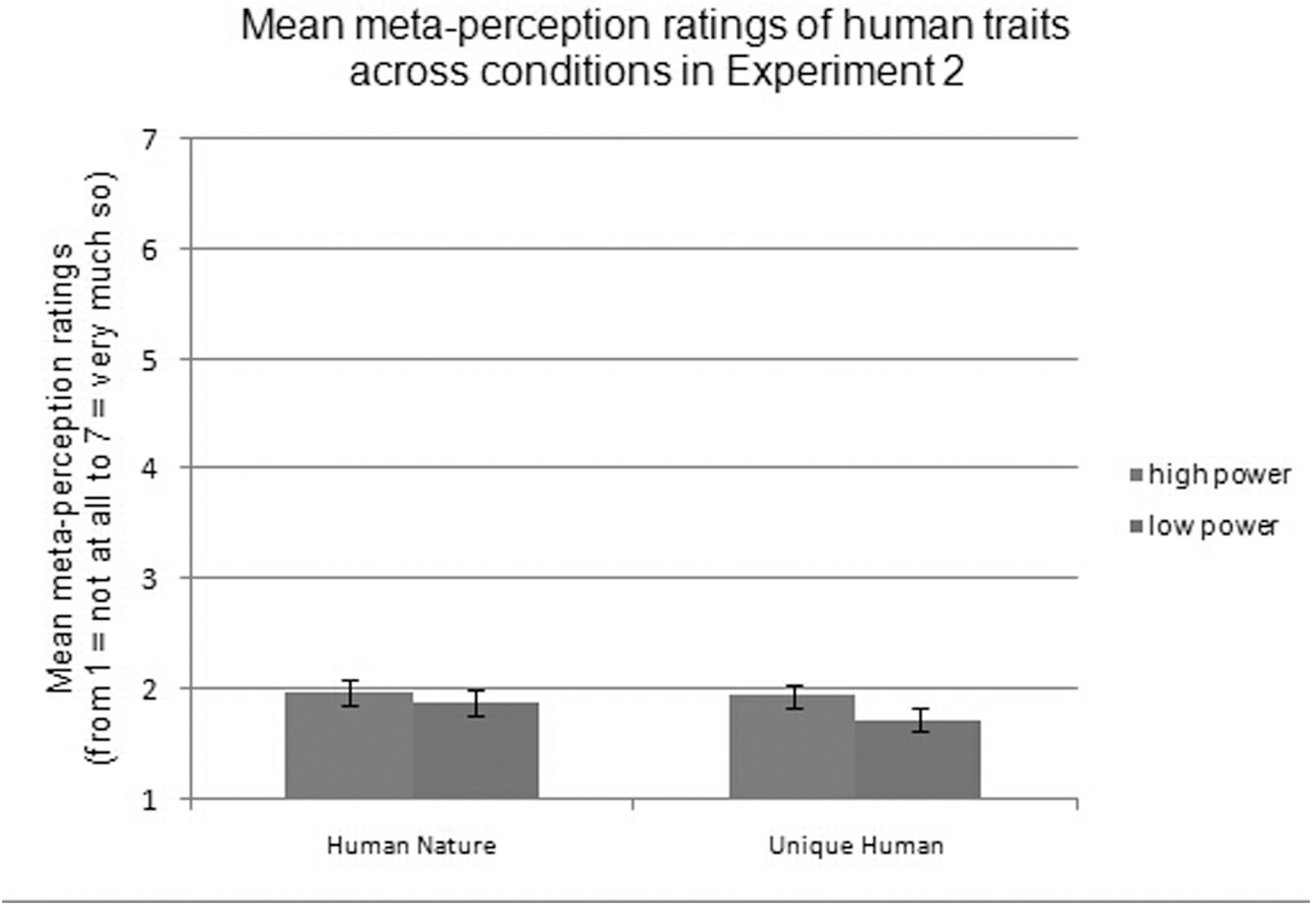
Mean meta-perception ratings of human traits across conditions in Experiment 2.

For the ratings of meta-perceptions, a significant 2 (power: high vs. low) × 2 (valence: positive vs. negative) × 2 (trait: HN vs. UH) interaction effect was not found, *F*(1, 77) = .25, *p* = .619. Then, separate 2 (power: high vs. low) × 2 (valence: positive vs. negative) repeated-measures ANCOVAs for each type of human trait were conducted. For HN traits, a main effect of valence was found, *F*(1, 77) = 11.07, *p* = .001, ƞ^2^ = .13, indicating that participants generally rated themselves as having more positive (*M =* 2.29, *SD* = 0.74) than negative (*M =* 1.56, *SD* = 0.71) HN traits. There was no main effect of power, *F*(1, 77) = 0.70, *p* = .404, demonstrating that participants who lacked power had equal levels of HN traits (*M =* 1.88, *SD* = 0.72) as those who had power (*M =* 1.97, *SD* = 0.72), as shown in Fig 3. Furthermore, there was an interaction between valence and power, *F*(1, 77) = 11.76, *p* = .001, ƞ^2^ = .13. Simple effects analysis showed that compared to those with power, participants who lacked power believed that others viewed them as having fewer positive HN traits but equal numbers of negative HN traits (positive: *F*(1,77) = 8.97, *p* = .004, ƞ^2^ = .10; negative: *F*(1,77) = 3.61, *p* = .061).

For UH traits, there was a main effect of valence, *F*(1, 77) = 12.62, *p* = .001, ƞ^2^ = .14, with participants rating themselves as having more positive (*M =* 2.21, *SD* = 0.70) than negative (*M =* 1.48, *SD* = 0.56) UH traits. There was also an effect of power, *F*(1, 77) = 6.05, *p* = .016, ƞ^2^ = .07, demonstrating that participants who lacked power viewed themselves as having fewer UH traits (*M =* 1.73, *SD* = 0.64) than did those who had power (*M =* 1.95, *SD* = 0.64), as shown in Fig 3. This supports our hypothesis concerning the importance of power in the self-perceived UH traits. Furthermore, valence interacted with power, *F*(1, 77) = 18.77, *p <* .001, ƞ^2^ = .20, and simple effects analysis showed that compared to participants who had power, participants who lacked power believed that others viewed them as having fewer positive UH traits but equal numbers of negative UH traits (positive: *F*(1,77) = 19.12, *p <* .001, ƞ^2^ = .20; negative: *F*(1,77) = 2.16, *p* = .15).

### Discussion

Experiment 2 replicated the basic effect found in Experiment 1 using a different methodology and supported the effect of power on HN traits in self-perception; specifically, the power × valence interaction in this study replicated the finding in Experiment 1 that powerlessness diminishes positive human traits. However, more importantly, the results highlighted the central role of UH traits in meta-perception. In particular, subordinate participants were led to believe that the leader participants who exerted power over them saw them as lacking in UH traits. This finding may reflect links between the HN traits and self-perception and between the UH traits and meta-perception. Moreover, Experiment 2 shows that while powerlessness does not necessarily cause people to see themselves more negatively, it does affect self-perceived humanity.

However, Experiment 2 also left some questions unanswered. First, participants’ self-perceived human qualities may have been affected by specific stereotypes regarding the leader and subordinate roles rather than being caused by an actual sense of power. Second, as a perceived powerlessness leads to dehumanization in meta-perception, the possible effect of observers is of interest. If the effect of powerlessness were as aversive as it was observed to be, it may be relevant not only to the meta-perception of those involved in the power dynamic but also to the meta-perceptions of observers not involved in a power dynamic. Third, in the previous two experiments, we had no ratings from a control condition with which to compare our baseline ratings; therefore, it is not clear whose perception was affected by the power manipulation: the powerful, the powerless, or both. Therefore, Experiment 3 investigates whether individuals’ self-perceived humanity is negatively affected by powerlessness, or positively affected by powerfulness.

## Experiment 3

In this experiment, we aimed to conceptually replicate and extend our findings from Experiments l and 2 by adapting a more implicit means of priming the concept of power [24] and extending the self-dehumanization effect found in meta-perception to observers. In addition, we added a control group and made no predictions regarding whether there would be differences between the control, high-power, and low-power conditions.

### Method

#### Ethics Statement

The experiment was conducted after obtaining Institutional Review Board approval from the School of Psychology at Beijing Normal University. All participants gave informed written consent before testing began.

#### Participants

Two hundred fifty adults (96 male) received 15 RMB in return for their participation in this experiment. Their ages ranged from 21 to 53 years (*M =* 30.43, *SD =* 2.88).

#### Procedure

*Power manipulation*. We conducted Experiment 3 in an office environment using a procedure adapted from Chen and colleagues [39] and Carney, Cuddy, and Yap [40]. Participants in the experimental condition entered the office in pairs and were randomly assigned to one of the two power conditions (high and low). They were then asked to either sit in a boss’s chair behind a desk and display an open posture, or sit in a guest’s chair in front of the desk and display a constrained posture. The control participants entered another room in pairs and both sat in normal chairs of the same style. We also ensured that participants were of the same sex in every interaction, and that they did not talk to each other during the course of the experiment.

*Dehumanization traits*. After the power manipulation, participants were instructed to complete an ostensibly unrelated task, rating themselves and the meta-perceptions of the observers on the same measures of humanity used in Experiment 2, while seated in the boss’s chair (high-power condition), the guest chair (low-power condition), or the non-descript chairs (control condition). For the meta-perception ratings, the item stem was as follows: “I felt like the observers considered me as....”

*Affect*. Next, participants completed the Positive and Negative Affect Schedule [41] using a 5-point scale (1 = not at all to 5 = very much so) to rate how each adjective described how they felt at that particular moment. The scores of items were averaged to form the scale scores.

*Manipulation check*. At the end of the procedure, participants completed a questionnaire and provided ratings for their sense of power using two items adapted from Kraus and colleagues [42], including “Right now, I feel I have a great deal of power” and “Right now, I feel like my wishes don’t carry much weight” [reversed]. Both items were measured on a 7-point Likert scale ranging from 1 = strongly disagree to 7 = strongly agree.

The process lasted approximately 20 minutes. Participants were then probed as to the purpose of the study, debriefed, and thanked individually.

### Results

Ten participants were excluded. Six did not complete the questionnaire, and two expressed some suspicion regarding the purpose of the experiment. In order to maintain same-sex dyads, we eliminated two other participants. The analyses were conducted using the data from the remaining 240 participants.

#### Manipulation check

Answers to the manipulation check items were reliable (Cronbach’s *α* = .88). They were averaged to form an overall sense of power score, and as expected, participants in the different conditions exhibited different levels of power, *F*(2, 239) = 69.97, *p <* .001, ƞ^2^ = .24. Specifically, participants more often described themselves as being powerful in the high-power condition (*M =* 5.22, *SD =* 1.04) relative to participants in the control condition (*M =* 4.04, *SD =* 0.92), *t*(158) = 6.98, *p <* .001. Similarly, participants described themselves as more powerless in the low-power condition relative to participants in the control condition (*M =* 3.52, *SD =* 1.21), *t*(158) = 5.08, *p <* .001.

#### Affect

The ratings for the affect items did not differ between the conditions, *F*s < 1; therefore, they will not be discussed further.

#### Dehumanization traits

The measures of human traits were found to be reliable (self-perception, HN: Cronbach’s *α* = .73, UH: Cronbach’s *α* = .70; meta-perception of observers, HN: Cronbach’s *α* = .75, UH: Cronbach’s *α* = .70). As in Experiment 2, ANCOVAs were used to explore ratings for self-perception and meta-perception of observers, with power condition (high vs. control vs. low) as the between-subjects factor, while valence (positive vs. negative) and trait (HN vs. UH) as within-subjects factors. We also entered participants’ gender as covariates, as in Experiments 1 and 2.

**Fig 4:**
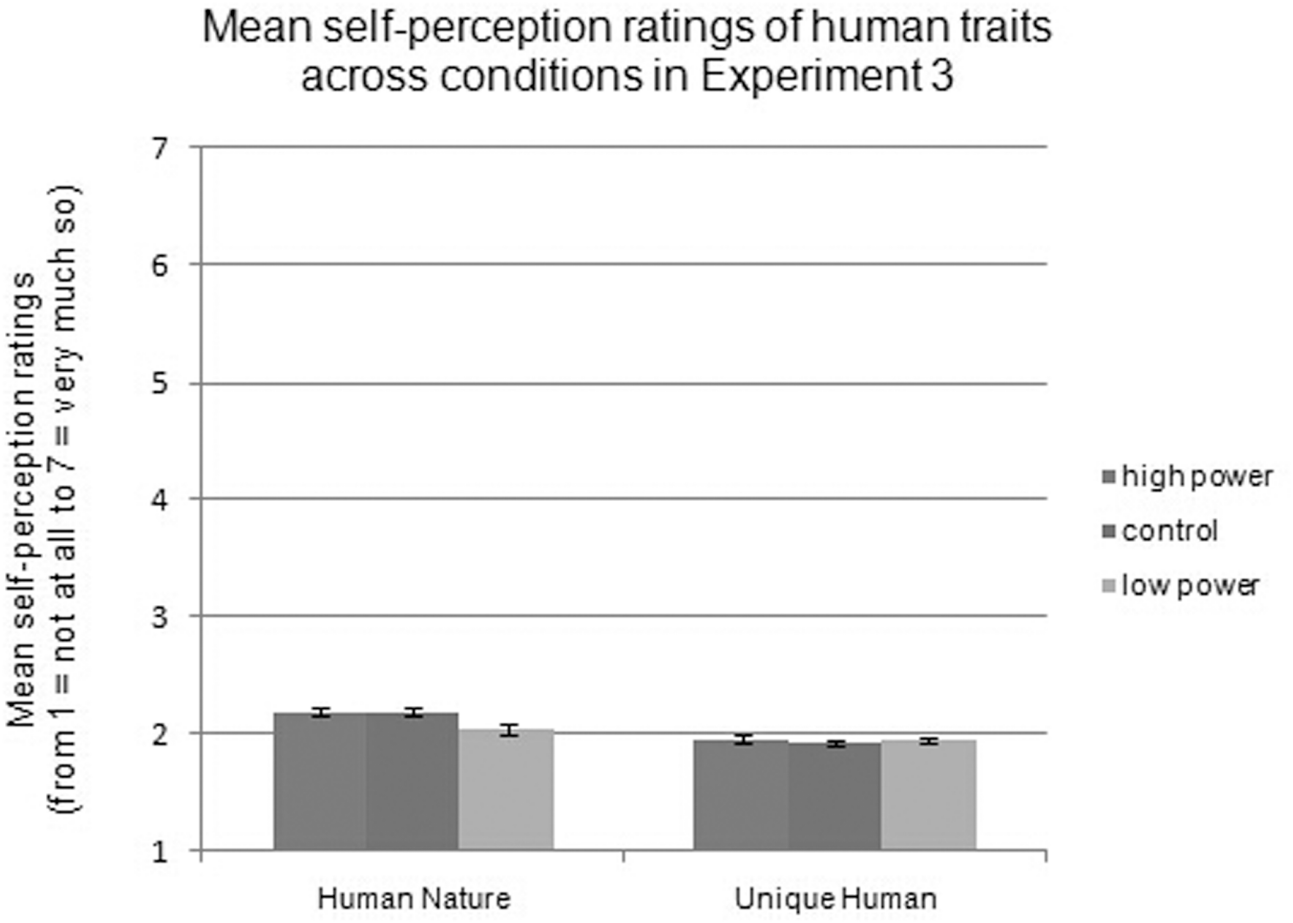
Mean self-perception ratings of human traits across conditions in Experiment 3.

Regarding self-perception, a significant 3 (power: high vs. control vs. low) × 2 (valence: positive vs. negative) × 2 (trait: HN vs. UH) interaction effect was not found, *F*(1, 77) = 1.66, *p* = .193. Then, separate 2 (power: high vs. low) × 2 (valence: positive vs. negative) repeated-measures ANCOVAs for each type of human trait was conducted. For HN traits, a main effect of valence was found, *F*(1, 236) = 61.04, *p <* .001, ƞ^2^ = .21, indicating that participants rated themselves as having more positive (*M =* 2.65, *SD* = 0.24) than negative (*M =* 1.63, *SD* = 0.33) HN traits. A main effect of power was also found, *F*(2, 236) = 5.82, *p* = .003, ƞ^2^ = .05, indicating that—independent of valence—participants’ ratings of HN traits differed across power conditions. Specifically, participants in the low-power condition (*M =* 2.04) viewed themselves as having fewer HN traits than did participants in the control (*M =* 2.19, *p* = .003) and high-power (*M =* 2.19, *p* = .004) conditions. The control and high-power conditions did not differ from each other, *p* = .989, as shown in Fig 4. Once again, valence interacted with power, *F*(2, 236) = 27.55, *p <* .001, ƞ^2^ = .19. Simple effects analysis showed that for positive HN traits, the differences among the power conditions were significant, *F*(2, 236) = 42.97, *p* < .001, ƞ^2^ = .27. Participants in the low-power condition (*M =* 2.35) viewed themselves as having fewer positive HN traits than did participants in the control (*M =* 2.71, *p* < .001) and high-power (*M =* 2.89, *p* < .001) conditions. The control and high-power conditions also significantly differed from each other, *p* = .002. For negative HN traits, the differences among the power conditions were significant, *F*(2, 236) = 4.62, *p* = .011, ƞ^2^ = .04. Participants in the high-power condition (*M =* 1.50) viewed themselves as having fewer negative HN traits than did participants in the low-power (*M* = 1.72, *p* = .004) and control (*M* = 1.67, *p =* .031) conditions. The low-power and control conditions did not differ from each other, *p* = .448.

For the UH dimension, a main effect of valence, *F*(1, 236) = 95.58, *p <* .001, ƞ^2^ = .29 revealed that participants rated themselves as having more positive (*M =* 2.71, *SD* = 0.22) than negative (*M* = 1.19, *SD* = 0.24) UH traits. However, UH trait ratings did not differ across power conditions (*M*_high-power_ = 1.96, *M*_control_ = 1.93, *M*_low-power_ = 1.95), *F*(2, 236) = 0.46, *p* = .632, as shown in Fig 4. Valence interacted with power, *F*(2, 236) = 28.46, *p <* .01, ƞ^2^ = .19. Simple effects analysis showed that there were differences among the conditions for positive UH traits, *F*(2, 236) = 20.13, *p* < .001, ƞ^2^ = .15. Specifically, participants in the low-power condition (*M* = 2.52) viewed themselves as having fewer positive UH traits than did participants in the control (*M =* 2.74, *p* < .001) and high-power (*M =* 2.87, *p* < .001) conditions. The control and high-power conditions also differed from each other, *p* = .009. There were also differences among the power conditions for negative UH traits, *F*(2, 236) = 19.15, *p* < .001, ƞ^2^ = .14. Participants in the low-power condition (*M* = 1.40) viewed themselves as having more negative UH traits than did participants in the control (*M* = 1.12, *p* < .001) and high-power (*M =* 1.04, *p* < .021) conditions. The control and high-power conditions did not differ from each other, *p* = .188.

**Fig 5:**
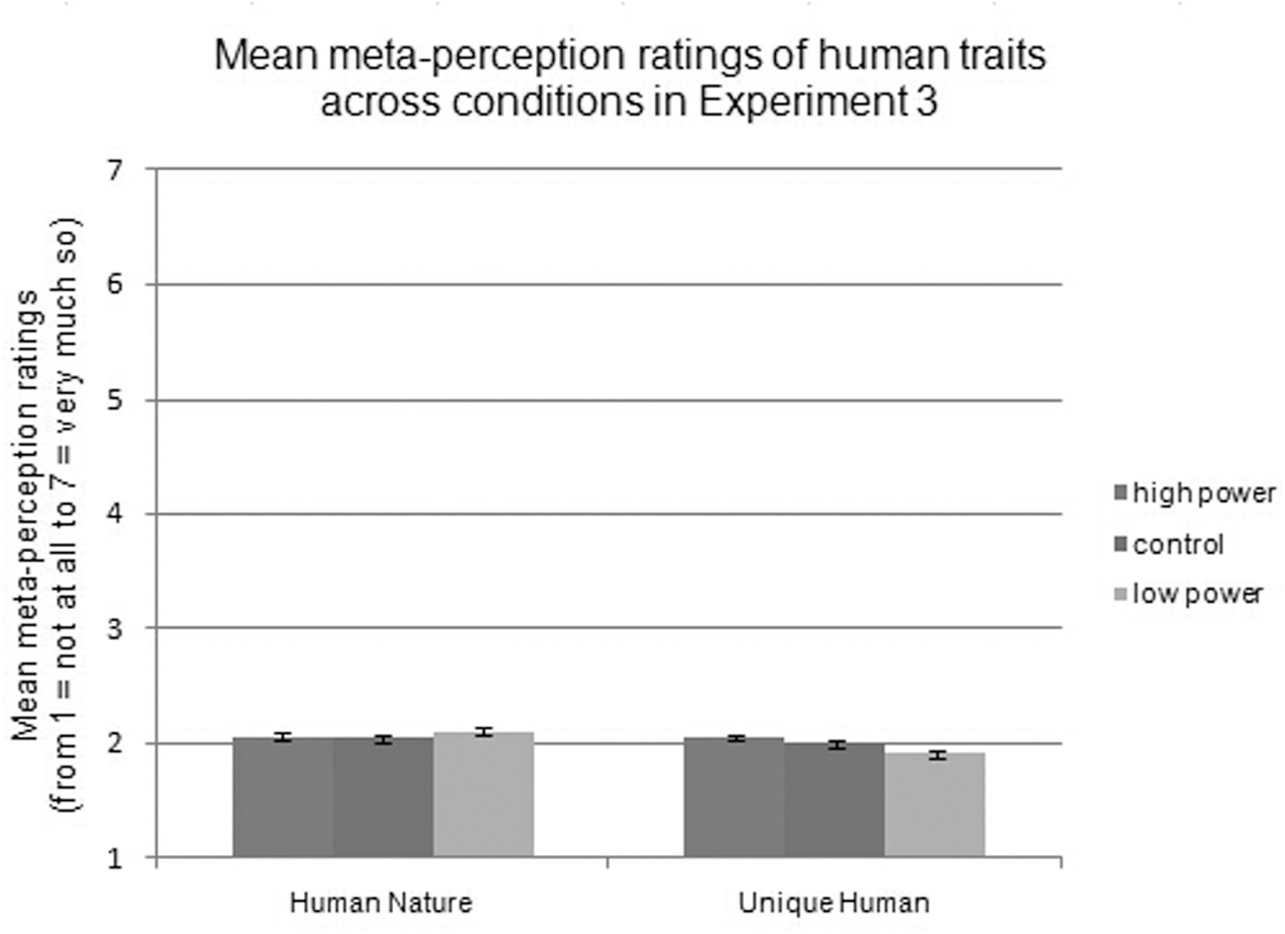
Mean meta-perception ratings of human traits across conditions in Experiment 3.

Concerning the ratings of meta-perceptions of observers, a 3 (power: high vs. control vs. low) × 2 (valence: positive vs. negative) × 2 (trait: HN vs. UH) interaction effect was not found, *F*(1, 77) = .23, *p* = .795. Then, separate 3 (power: high vs. low vs. control) × 2 (valence: positive vs. negative) repeated-measures ANCOVAs for each type of human trait were conducted. For HN traits, a main effect of valence was found, *F*(1, 236) = 40.15, *p <* .001, ƞ^2^ = .15, indicating that participants rated themselves as having more positive (*M =* 2.62, *SD* = 0.21) than negative (*M =* 1.53, *SD* = 0.31) HN traits. However, no main effect of power was found (*M*_low-power_ = 2.06; *M*_control_ = 2.05; *M*_high-power_ = 2.10), *F*(2, 236) = .73, *p* = .482, as shown in Fig 5. There was an interaction between valence and power, *F*(2, 236) = 20.67, *p <* .001, ƞ^2^ = .15, with simple effects showing that for positive HN traits, there were differences among the power conditions, *F*(2, 236) = 24.70, *p* < .001, ƞ^2^ = .17. Specifically, participants in the low-power condition (*M* = 2.44) viewed themselves as having fewer positive HN traits than did participants in the control (*M =* 2.60, *p* = .006) and high-power (*M =* 2.82, *p* < .001) conditions. The control and high-power conditions also differed from each other, *p* < .001. Additionally, there were differences among the power conditions for negative HN traits, *F*(2, 236) = 7.28, *p* = .001, ƞ^2^ = .06. Participants in the low-power condition (*M* = 1.68) viewed themselves as having more negative HN traits than did participants in the control (*M* = 1.51, *p* = .029) and high-power (*M =* 1.39, *p* < .001) conditions. The control and high-power conditions did not differ from each other, *p* = .102.

For UH traits, there was again a robust effect of valence, *F*(1, 236) = 67.57, *p <* .001, ƞ^2^ = .22, indicating that participants thought they were viewed as having more positive (*M =* 2.54) than negative (*M =* 1.44) UH traits. There was also an effect of power, *F*(2, 236) = 8.40, *p <* 0.001, ƞ^2^ = .07. Specifically, participants in the low-power condition (*M =* 1.91) viewed themselves as having fewer UH traits than did participants in the control (*M =* 2.00, *p* = .011) and high-power (*M =* 2.05, *p* < .001) conditions. The control and high-power conditions did not differ from each other, *p* = .130, as shown in Fig 5. Again, valence interacted with power, *F*(2, 236) = 33.18, *p <* .001, ƞ^2^ = .22. Simple effects analysis showed that there were differences between power conditions for positive UH traits, *F*(2, 236) = 37.53, *p* < .001, ƞ^2^ = .24. Participants in the low-power condition (*M* = 2.28) viewed themselves as having fewer positive UH traits than did participants in the control (*M =* 2.56, *p* < .001) and high-power (*M =* 2.78, *p* < .001) conditions. The control and high-power conditions also differed from each other, *p* < .001. There were also differences among the power conditions for negative UH traits, *F*(2, 236) = 6.71, *p* = .001, ƞ^2^ = .05. Participants in the low-power condition (*M =* 1.54) viewed themselves as having more negative UH traits than did participants in the control (*M* = 1.44, *p* = .046) and high-power (*M =* 1.33, *p* < .001) conditions. The control and high-power conditions did not differ from each other, *p* = .094.

### Discussion

Recent research has shown that power effects are so ubiquitous that they can have a psychological impact even outside an individual’s awareness (likely by activating power’s mental representation; see Smith and Galinsky [43]). Using a different methodology, Experiment 3 provided a more robust link between the HN traits and self-perception, as well as between the UH traits and meta-perception.

More importantly, these results show that the self-dehumanization effect only occurred among low-power individuals. There were no differences observed between the high-power and control conditions. In the control condition, participants just performed a free-will task, which may have given them a sense of control. This might explain why the effects of the high-power and control conditions did not differ. Meanwhile, previous studies have shown that powerful individuals show a greater number of human traits, such as agency and cognitive flexibility [5, 44], perhaps because the human traits examined in previous studies have been positive. This is consistent with the current finding that individuals in the high-power condition attributed more positive human traits to themselves compared to those in the control condition.

## General Discussion

In all three experiments, we used different power manipulations and measures of perceived humanity to provide empirical evidence for our notion of the self-dehumanizing consequences of perceived powerlessness. First, people in low-power positions viewed themselves as less human than did those in high-power positions, and the effects occurred even within an implied power situation with no clear power cues. Second, as predicted, HN and UH traits were both relevant dimensions of self-dehumanization. More importantly, these two dimensions played different roles in self-dehumanization when powerlessness was experienced. Specially, a decrease in power induces the HN traits of self-dehumanization in self-perception and the UH traits in meta-perceptions. Third, the powerless believed they were viewed as less human not only by people in the power dynamic (the powerful) but also the outside observers. Moreover, all of the results were supported after controlling for mood and the valence of dehumanization traits. That is, self-dehumanization was a consequence of powerless rather than an incidental result of a change in mood or negative self-view. Thus, this study demonstrates that experiencing powerless shapes self-dehumanization and is an important extension of theories of power and dehumanization.

### Implications for Power Research

This research extends previous knowledge regarding powerlessness by providing evidence that experiencing powerless has implications for our self-perception and meta-perception of human qualities. Powerless individuals express an indifference to subjectivity (self-dehumanization in its extreme form, particularly regarding HN traits), which may explain the tendency towards inhibition when powerless is experienced. Our work extends this view by demonstrating the consequences of the denial of HN traits on the self-perception of powerless individuals. Meanwhile, our findings indicate that powerless individuals see themselves as lacking in qualities that distinguish humans from other animals in meta-perception. Importantly, this is consistent with the finding that powerful individuals dehumanize powerless individuals [18, 19], implying that powerless individuals not only pay closer attention to the state of power but also have a self-view that mirrors that of the powerful individual. Furthermore, the same effect occurs with respect to observers. This is consistent with the notion that an individual experiencing powerless is more sensitive towards others [45] and pays more attention to social contexts [46]. Our work extends this view by demonstrating the consequences of the denial of UH traits on the meta-perception of powerless individuals.

This research also augments our knowledge of the stability of power hierarchies [47]; such hierarchies can fundamentally alter one’s self-perception of humanity and may thus be important contributors to powerless individuals’ psychological states.

### Implications for Dehumanization Research

Early research emphasized the fact that dehumanization processes typically occur in the context of immorality, aggression, or extremity. Recent work, however, has found that dehumanizing others can be conceptualized as a subtle daily phenomenon [21, 48]. Our findings broaden the domain of self-dehumanization by providing important reflections on the role of ordinary interpersonal relationships in the perception of our own humanity. Daily engagements in disadvantageous relationships (e.g., in the low-power position) appear to be sufficient to cause us to perceive ourselves as possessing fewer human qualities.

It is important to note that our results provide a model that suggests that self-dehumanization in its most subtle forms is associated with perceived powerlessness. This also supports the convenient two-dimensional framework [10] presented in previous studies on humanity [11, 49–51]. The results presented here support the notion that human nature plays a role in interpersonal forms of maltreatment [11, 14, 15]. Consistent with previous findings, we argue that individuals’ entry into states that deny their basic HN traits in positions of low power is an adaptive response designed to cope with uncertainty and threats in the social environment [14]. Without the capacity for self-dehumanization, people would view themselves as human in the way that they perceive powerful individuals: as sensitive to pain and willing to obtain agency. Therefore, they would experience pain and depression and would be unable to endure such environments. In short, the mechanical state affords powerless individuals the psychological benefits of avoiding self-awareness and self-will [14].

Moreover, meta-perception refers to the ability to view oneself from the perspective of others. Our results support that UH traits play a role in powerless individuals’ meta-perceptions of those involved in the power dynamic. This is congruent with the theory that denial of UH traits lays an important role in power [18, 19]. We argue that powerless individuals who view themselves as possessing fewer UH traits may add value to their relationships, as self-dehumanization makes it easier for powerless individuals to establish and maintain relationships with others. More importantly, previous research has investigated the observer effects on targets’ action [52], but the empirical evidence in support of observer effects on dehumanization is rare (but see [14]). As far as we are aware, the current study is the first to test dehumanization directly from the perspective of the meta-perception of observers not involved in a power dynamic. In observers’ eyes, the powerless are likely to have few UH traits since the powerless have few or no opportunities to express these traits to others. Our work supports the notion that the powerless perceive themselves through the eyes’ of the powerful similarly to how observers perceive them—that is, as possessing few UH traits.

Our work also provides important insights into the link between power and dehumanization. Previous research has traditionally demonstrated various means of dehumanization related to the perception of others [11, 50, 53] and other groups [13, 49, 51, 54]. Our findings support previous research in which dehumanization was experienced via the self-concepts of perpetrator [15] and victim [14], indicating that people self-dehumanize in response to a perceived powerless.

### Limitations and Future Directions

The present study was theoretically motivated, and the findings were consistent with those of previous studies. However, there were some limitations, and these will guide the direction of future research. First, these experiments focused exclusively on recall procedures and simulated situations; thus, this research should be extended to more interpersonal, unequal, and ecologically valid social power contexts such as social class, status, gender, and race. Second, it should be noted that only explicit measures of dehumanization were used, allowing direct access to participants’ self-perception of humanity. Although this provided a good demonstration of self-dehumanization effects as a result of power priming, it is not clear whether this self-dehumanizing orientation would extend to implicit methods; for instance, whether self-dehumanization would be evident in implicit self-concepts, using methodologies such as the Implicit Association Test or the Go/No-Go Association Test. Third, to the best of our knowledge, this study was the first to investigate how self-dehumanization was involved in powerlessness. All of our participants were Chinese, and previous studies have shown that Chinese participants differ from Western participants in the contribution of HN and UH traits [49, 55, 56]. Therefore, future research should investigate cultural differences in self-dehumanization of the powerless. Perhaps more interestingly, previous research has shown that illegitimate or unstable powerlessness can reverse the effects of powerlessness, as illegitimacy disinhibits powerless individuals [57], enhances their self-regulation in goal pursuit, and promotes greater action orientation and cognitive flexibility than is evidenced among powerful individuals [58, 59]. Instability also makes powerless individuals think more creatively [60]. Therefore, a worthy avenue for future research would be to investigate whether illegitimate or unstable powerlessness serves to eliminate self-dehumanization.
